# Crystal structure of 4,4′-(ethane-1,2-di­yl)bis­(2,6-di­bromo­aniline)

**DOI:** 10.1107/S2056989014027182

**Published:** 2015-01-01

**Authors:** Ines Hauptvogel, Wilhelm Seichter, Edwin Weber

**Affiliations:** a Institut für Organische Chemie, TU Bergakademie Freiberg, Leipziger Strasse 29, D-09596 Freiberg/Sachsen, Germany

**Keywords:** crystal structure, 4,4′-(ethane-1,2-di­yl)bis­(2,6-di­bromo­aniline), framework structures

## Abstract

In the title compound, C_14_H_12_Br_4_N_2_, the mol­ecule lies across an inversion center and hence the benzene rings are strictly coplanar. In the crystal, mol­ecules are linked by N—H⋯N and weak N—H⋯Br hydrogen bonds, forming a two-dimensional network parallel to (101). In addition, type II Br⋯Br inter­actions [3.625 (4) Å] complete a three-dimensional supra­molecular network.

## Abstract 2

The 4,4′-(ethane-1,2-di­yl)bis­(2,6-di­bromo­aniline) mol­ecule lies across an inversion center and hence the benzene rings are strictly planar. Mol­ecules are linked by N—H⋯N and weak N—H⋯Br hydrogen bonds, forming a two-dimensional network parallel to (101). Type II Br⋯Br inter­actions complete a three-dimensional supra­molecular network.

## Chemical context   

Spacer-type compounds are vital for the generation of a variety of framework structures including metal organic (MOF) (MacGillivray, 2010 ▸), hydrogen-bonded (HBN) (Elemans *et al.*, 2009 ▸) or covalent organic (COF) (El-Kaden *et al.*, 2007 ▸) network species. The title compound is an inter­mediate substance of a corresponding synthesis of a corresponding spacer molecule. Moreover, tecton-like mol­ecules having terminally attached inter­acting sites are inter­esting building blocks in the field of organic crystal engineering (Tiekink *et al.*, 2010 ▸), in particular involving potentially competitive groups, in itself forming hydrogen bonds (Braga & Crepioni, 2004 ▸) or halogen contacts (Awwadi *et al.*, 2006 ▸; Metrangolo & Resnati, 2008 ▸) by preference in the crystal state. Such a test case is given with the oligo­bromo­amino-containing title compound.
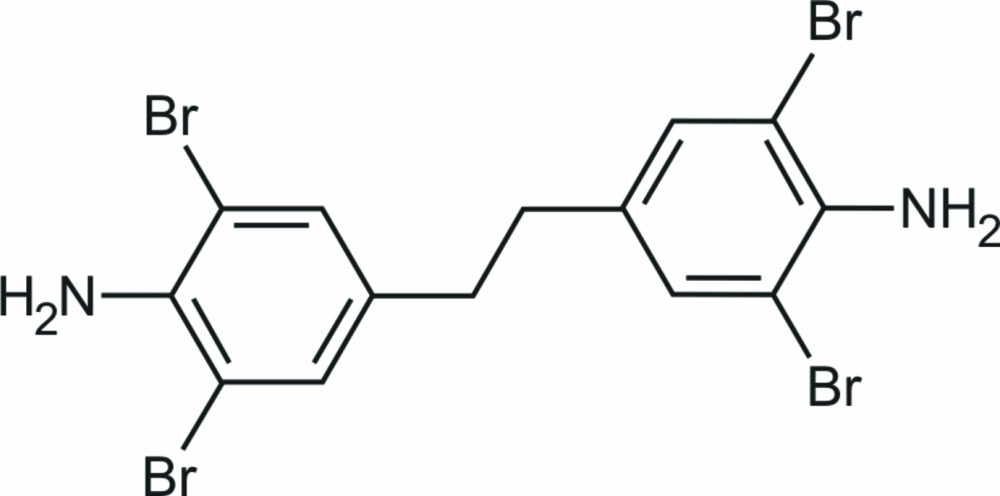


## Structural commentary   

**Figure 1:**
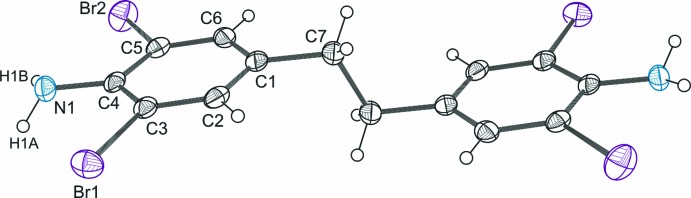
The mol­ecular structure of the title compound with displacement ellipsoids for non-H atoms drawn at the 50% probability level. Unlabeled atoms are related by the symmetry operator (−*x* + 1, −*y* + 2, −*z*).

The title mol­ecule lies across an inversion center and hence the benzene rings are strictly coplanar (Fig. 1 ▸). The conformation of the mol­ecular backbone agrees well with those found in the structure of 1,2-bi­phenyl­ethane (Harada & Ogawa, 2001 ▸) and a great number of its ring-substituted derivatives (Kahr *et al.*, 1995 ▸; Moorthy *et al.*, 2005 ▸). The C*sp* ^3^—C*sp* ^3^ and C*sp* ^3^—C*sp* ^2^ bond lengths of 1.535 (6) and 1.514 (4) Å are in the normal range.

## Supra­molecular features   

**Figure 2:**
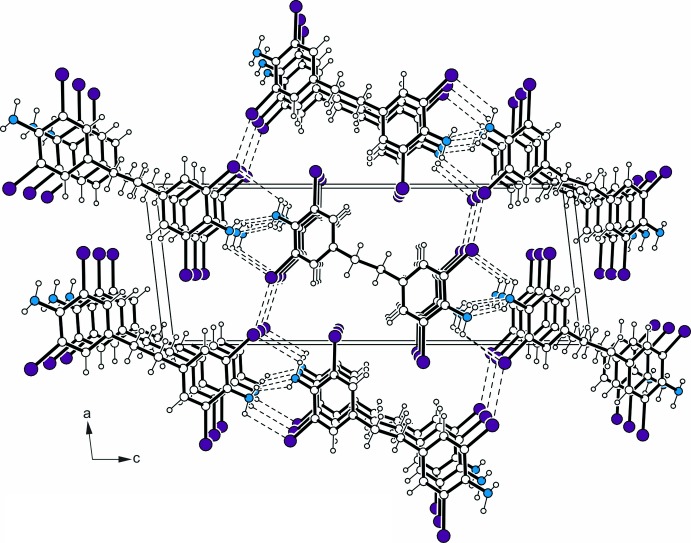
Part of the crystal structure viewed along the *b* axis. N atoms are displayed as blue and Br atoms as violet circles. Hydrogen bonds and Br⋯Br contacts are shown as dashed lines.

The amino group hydrogen atoms take part in mol­ecular association (Table 1 ▸) by forming conventional N—H⋯N hydrogen bonds (Jeffrey, 1997 ▸, see Table 1 ▸) and weak N—H⋯Br contacts (Desiraju & Steiner, 1999 ▸) resulting in the formation of a layer structure parallel to (101) (Fig. 2 ▸). Inter­layer association is accomplished by type II, Br⋯Br contacts [3.625 (4) Å, θ_1_ = 109.7 (2), θ_2_ = 150.7 (2)°] (Awwadi *et al.*, 2006 ▸; Metrangolo & Resnati, 2008 ▸).

## Synthesis and crystallization   

In an imitation of a described procedure (Berger *et al.*, 1998 ▸) preparation of the title compound was achieved by a bromination reaction of a solution of 4,4′-di­amino­biphenyl (10.0 g, 47.14 mmol) in glacial acetic acid (760 ml) using bromine (30.3 g, 0.19 mol, dissolved in 40 ml glacial acetic acid). After having stirred for 2 h at room temperature, water was added to the mixture. The raw product which precipitated was collected, washed with water and treated with boiling glacial acetic acid to yield 19.6 g (79%) of a greenish powder. Slow crystallization from toluene gave colourless needles of the title compound suitable for X-ray structural analysis. M.p. >593 K. IR (KBr) 3329, 3190, 3033, 2940, 2915, 2851, 1617, 1581, 1542, 1486, 1060, 892, 871. MS (EI) *m*/*z*: found – 527.5; calculated for C_14_H_12_N_2_Br_4_ – 527.87. Elemental analysis: found – C 31.53, H 2.34, N 5.59; calculated for C_14_H_12_N_2_Br_4_ – C 31.85, H 2.29, N 5.31. 4,4′-Di­amino­bibenzyl was purchased (Sigma–Aldrich). The melting point was measured on a hot-stage microscope (Rapido Dresden). IR and mass (EI–MS) spectra were performed using Nicolet 510 FTIR and Finnigan Mat 8200 instruments, respectively.

## Refinement   

Crystal data, data collection and structure refinement details are summarized in Table 2 ▸. C-bound H atoms were positioned geometrically (C—H = 0.93 Å for aromatic and C—H 0.97 Å for methyl­ene H) and refined using a riding model with *U*_iso_(H) = 1.2 *U*_eq_(C). The amino H atoms were located in a Fourier map and the N—H distances restrained to 0.89 (1) Å.

## Supplementary Material

Crystal structure: contains datablock(s) I, New_Global_Publ_Block. DOI: 10.1107/S2056989014027182/lh5742sup1.cif

Structure factors: contains datablock(s) I. DOI: 10.1107/S2056989014027182/lh5742Isup2.hkl

Supporting information file. DOI: 10.1107/S2056989014027182/lh5742Isup3.cml

CCDC reference: 1038844

Additional supporting information:  crystallographic information; 3D view; checkCIF report

## Figures and Tables

**Table 1:** Hydrogen-bond geometry (, )

*D*H*A*	*D*H	H*A*	*D* *A*	*D*H*A*
N1H1*A*N1^i^	0.88(2)	2.45(3)	3.206(4)	145(2)
N1H1*B*Br1^i^	0.88(2)	3.03(3)	3.521(4)	117(2)

Symmetry code: (i) 
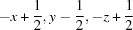
.

**Table 2:** Experimental details

Crystal data	
Chemical formula	C_14_H_12_Br_4_N_2_
*M*_r_	527.86
Crystal system, space group	Monoclinic, *P*2_1_/*n*
Temperature (K)	153
*a*, *b*, *c* ()	8.1219(4), 4.4962(2), 21.5327(9)
()	96.706(3)
*V* (^3^)	780.95(6)
*Z*	2
Radiation type	Mo *K*
(mm^1^)	10.30
Crystal size (mm)	0.30 0.20 0.08

Data collection	
Diffractometer	Bruker APEXII CCD area detector
Absorption correction	Multi-scan (*SADABS*; Bruker, 2007 ▸)
*T*_min_, *T*_max_	0.148, 0.493
No. of measured, independent and observed [*I* > 2(*I*)] reflections	6211, 1356, 1213
*R*_int_	0.034
(sin /)_max_ (^1^)	0.597

Refinement	
*R*[*F* ^2^ > 2(*F* ^2^)], *wR*(*F* ^2^), *S*	0.023, 0.057, 1.05
No. of reflections	1356
No. of parameters	99
No. of restraints	2
H-atom treatment	H atoms treated by a mixture of independent and constrained refinement
_max_, _min_ (e ^3^)	0.52, 0.29

Computer programs: *APEX2* and *SAINT* (Bruker, 2007 ▸), *SHELXS97*, *SHELXL97* and *SHELXTL* (Sheldrick, 2008 ▸), *ORTEP-3 for Windows* (Farrugia, 2012 ▸).

## supplementary crystallographic information

### Crystal data

**Table:**

C_14_H_12_Br_4_N_2_	*F*(000) = 500
*M**_r_* = 527.86	*D*_x_ = 2.245 Mg m^−^^3^
Monoclinic, *P*2_1_/*n*	Mo *K*α radiation, λ = 0.71073 Å
Hall symbol: -P 2yn	Cell parameters from 3117 reflections
*a* = 8.1219 (4) Å	θ = 2.6–26.4°
*b* = 4.4962 (2) Å	µ = 10.30 mm^−^^1^
*c* = 21.5327 (9) Å	*T* = 153 K
β = 96.706 (3)°	Needle, colourless
*V* = 780.95 (6) Å^3^	0.30 × 0.20 × 0.08 mm
*Z* = 2	

### Data collection

**Table:**

Bruker APEXII CCD area-detector diffractometer	1356 independent reflections
Radiation source: fine-focus sealed tube	1213 reflections with *I* > 2σ(*I*)
Graphite monochromator	*R*_int_ = 0.034
φ and ω scans	θ_max_ = 25.1°, θ_min_ = 2.6°
Absorption correction: multi-scan (*SADABS*; Bruker, 2007)	*h* = −9→9
*T*_min_ = 0.148, *T*_max_ = 0.493	*k* = −5→4
6211 measured reflections	*l* = −25→25

### Refinement

**Table:**

Refinement on *F*^2^	Primary atom site location: structure-invariant direct methods
Least-squares matrix: full	Secondary atom site location: difference Fourier map
*R*[*F*^2^ > 2σ(*F*^2^)] = 0.023	Hydrogen site location: inferred from neighbouring sites
*wR*(*F*^2^) = 0.057	H atoms treated by a mixture of independent and constrained refinement
*S* = 1.05	*w* = 1/[σ^2^(*F*_o_^2^) + (0.0287*P*)^2^ + 0.4643*P*] where *P* = (*F*_o_^2^ + 2*F*_c_^2^)/3
1356 reflections	(Δ/σ)_max_ < 0.001
99 parameters	Δρ_max_ = 0.52 e Å^−^^3^
2 restraints	Δρ_min_ = −0.29 e Å^−^^3^

### Special details

**Table:**

Geometry. All e.s.d.'s (except the e.s.d. in the dihedral angle between two l.s. planes) are estimated using the full covariance matrix. The cell e.s.d.'s are taken into account individually in the estimation of e.s.d.'s in distances, angles and torsion angles; correlations between e.s.d.'s in cell parameters are only used when they are defined by crystal symmetry. An approximate (isotropic) treatment of cell e.s.d.'s is used for estimating e.s.d.'s involving l.s. planes.
Refinement. Refinement of *F*^2^ against ALL reflections. The weighted *R*-factor *wR* and goodness of fit *S* are based on *F*^2^, conventional *R*-factors *R* are based on *F*, with *F* set to zero for negative *F*^2^. The threshold expression of *F*^2^ > σ(*F*^2^) is used only for calculating *R*-factors(gt) *etc*. and is not relevant to the choice of reflections for refinement. *R*-factors based on *F*^2^ are statistically about twice as large as those based on *F*, and *R*- factors based on ALL data will be even larger. The distances of N—H bonds were restrained to a target value of 0.89(0.01) Å.

### Fractional atomic coordinates and isotropic or equivalent isotropic displacement parameters (Å^2^)

**Table:**

	*x*	*y*	*z*	*U*_iso_*/*U*_eq_	
Br1	0.57978 (4)	0.36821 (8)	0.225361 (14)	0.03020 (13)	
Br2	−0.06304 (4)	0.52607 (9)	0.094219 (15)	0.03690 (14)	
N1	0.2009 (3)	0.2907 (7)	0.19806 (11)	0.0243 (6)	
H1A	0.260 (3)	0.140 (5)	0.2143 (13)	0.020 (9)*	
H1B	0.102 (2)	0.223 (8)	0.1841 (14)	0.033 (9)*	
C1	0.3975 (4)	0.8813 (7)	0.07282 (13)	0.0216 (7)	
C2	0.4981 (3)	0.7477 (7)	0.12150 (13)	0.0221 (7)	
H2	0.6107	0.7920	0.1274	0.026*	
C3	0.4334 (3)	0.5501 (7)	0.16131 (13)	0.0203 (7)	
C4	0.2654 (3)	0.4736 (7)	0.15549 (12)	0.0190 (7)	
C5	0.1679 (3)	0.6141 (7)	0.10623 (13)	0.0210 (7)	
C6	0.2309 (4)	0.8108 (7)	0.06593 (13)	0.0222 (7)	
H6	0.1604	0.8969	0.0338	0.027*	
C7	0.4705 (4)	1.0862 (8)	0.02740 (14)	0.0279 (7)	
H7A	0.5633	1.1936	0.0493	0.034*	
H7B	0.3875	1.2306	0.0113	0.034*	

### Atomic displacement parameters (Å^2^)

**Table:**

	*U*^11^	*U*^22^	*U*^33^	*U*^12^	*U*^13^	*U*^23^
Br1	0.02849 (19)	0.0311 (3)	0.02914 (19)	0.00445 (14)	−0.00444 (13)	−0.00097 (14)
Br2	0.02079 (19)	0.0507 (3)	0.0385 (2)	−0.00335 (15)	0.00032 (14)	0.00005 (17)
N1	0.0284 (14)	0.0220 (17)	0.0237 (13)	−0.0027 (12)	0.0073 (11)	0.0028 (13)
C1	0.0308 (16)	0.0158 (18)	0.0196 (14)	0.0001 (13)	0.0095 (12)	−0.0052 (13)
C2	0.0205 (14)	0.0208 (18)	0.0264 (15)	−0.0016 (13)	0.0089 (12)	−0.0068 (15)
C3	0.0232 (15)	0.0212 (19)	0.0171 (13)	0.0019 (13)	0.0044 (12)	−0.0044 (13)
C4	0.0234 (15)	0.0183 (18)	0.0165 (13)	−0.0006 (13)	0.0070 (12)	−0.0056 (13)
C5	0.0198 (14)	0.0220 (19)	0.0218 (14)	0.0003 (12)	0.0051 (11)	−0.0061 (14)
C6	0.0311 (16)	0.0189 (19)	0.0172 (13)	0.0041 (13)	0.0050 (12)	−0.0015 (13)
C7	0.0422 (19)	0.0171 (19)	0.0273 (16)	−0.0008 (15)	0.0159 (14)	−0.0017 (14)

### Geometric parameters (Å, º)

**Table:**

Br1—C3	1.898 (3)	C2—H2	0.9300
Br2—C5	1.905 (3)	C3—C4	1.398 (4)
N1—C4	1.380 (4)	C4—C5	1.398 (4)
N1—H1A	0.877 (10)	C5—C6	1.378 (4)
N1—H1B	0.879 (10)	C6—H6	0.9300
C1—C6	1.381 (4)	C7—C7^i^	1.535 (6)
C1—C2	1.388 (4)	C7—H7A	0.9700
C1—C7	1.514 (4)	C7—H7B	0.9700
C2—C3	1.381 (4)		
			
C4—N1—H1A	119.6 (19)	C3—C4—C5	114.7 (3)
C4—N1—H1B	113 (2)	C6—C5—C4	123.3 (3)
H1A—N1—H1B	108 (3)	C6—C5—Br2	118.6 (2)
C6—C1—C2	117.7 (3)	C4—C5—Br2	118.1 (2)
C6—C1—C7	121.5 (3)	C5—C6—C1	120.7 (3)
C2—C1—C7	120.7 (3)	C5—C6—H6	119.7
C3—C2—C1	120.9 (3)	C1—C6—H6	119.7
C3—C2—H2	119.5	C1—C7—C7^i^	111.7 (4)
C1—C2—H2	119.5	C1—C7—H7A	109.3
C2—C3—C4	122.8 (3)	C7^i^—C7—H7A	109.3
C2—C3—Br1	118.5 (2)	C1—C7—H7B	109.3
C4—C3—Br1	118.8 (2)	C7^i^—C7—H7B	109.3
N1—C4—C3	122.0 (3)	H7A—C7—H7B	107.9
N1—C4—C5	123.2 (3)		
			
C6—C1—C2—C3	0.4 (4)	C3—C4—C5—C6	0.5 (4)
C7—C1—C2—C3	−177.0 (3)	N1—C4—C5—Br2	−4.4 (4)
C1—C2—C3—C4	−0.2 (5)	C3—C4—C5—Br2	−179.9 (2)
C1—C2—C3—Br1	178.3 (2)	C4—C5—C6—C1	−0.4 (5)
C2—C3—C4—N1	−175.7 (3)	Br2—C5—C6—C1	180.0 (2)
Br1—C3—C4—N1	5.8 (4)	C2—C1—C6—C5	−0.1 (4)
C2—C3—C4—C5	−0.2 (4)	C7—C1—C6—C5	177.3 (3)
Br1—C3—C4—C5	−178.7 (2)	C6—C1—C7—C7^i^	−89.9 (4)
N1—C4—C5—C6	176.0 (3)	C2—C1—C7—C7^i^	87.4 (4)

Symmetry code: (i) −*x*+1, −*y*+2, −*z*.

### Hydrogen-bond geometry (Å, º)

**Table:**

*D*—H···*A*	*D*—H	H···*A*	*D*···*A*	*D*—H···*A*
N1—H1*A*···N1^ii^	0.88 (2)	2.45 (3)	3.206 (4)	145 (2)
N1—H1*B*···Br1^ii^	0.88 (2)	3.03 (3)	3.521 (4)	117 (2)

Symmetry code: (ii) −*x*+1/2, *y*−1/2, −*z*+1/2.
